# Semantic Memory and the Hippocampus: Revisiting, Reaffirming, and Extending the Reach of Their Critical Relationship

**DOI:** 10.3389/fnhum.2019.00471

**Published:** 2020-01-24

**Authors:** Melissa C. Duff, Natalie V. Covington, Caitlin Hilverman, Neal J. Cohen

**Affiliations:** ^1^Department of Hearing and Speech Sciences, Vanderbilt University Medical Center, Nashville, TN, United States; ^2^Department of Psychology, Beckman Institute, University of Illinois, Champaign, IL, United States

**Keywords:** semantic, episodic, memory, language, hippocampus, methods

## Abstract

Since Tulving proposed a distinction in memory between semantic and episodic memory, considerable effort has been directed towards understanding their similar and unique features. Of particular interest has been the extent to which semantic and episodic memory have a shared dependence on the hippocampus. In contrast to the definitive evidence for the link between hippocampus and episodic memory, the role of the hippocampus in semantic memory has been a topic of considerable debate. This debate stems, in part, from highly variable reports of new semantic memory learning in amnesia ranging from profound impairment to full preservation, and various degrees of deficit and ability in between. More recently, a number of significant advances in experimental methods have occurred, alongside new provocative data on the role of the hippocampus in semantic memory, making this an ideal moment to revisit this debate, to re-evaluate data, methods, and theories, and to synthesize new findings. In line with these advances, this review has two primary goals. First, we provide a historical lens with which to reevaluate and contextualize the literature on semantic memory and the hippocampus. The second goal of this review is to provide a synthesis of new findings on the role of the hippocampus and semantic memory. With the perspective of time and this critical review, we arrive at the interpretation that the hippocampus does indeed make necessary contributions to semantic memory. We argue that semantic memory, like episodic memory, is a highly flexible, (re)constructive, relational and multimodal system, and that there is value in developing methods and materials that fully capture this depth and richness to facilitate comparisons to episodic memory. Such efforts will be critical in addressing questions regarding the cognitive and neural (inter)dependencies among forms of memory, and the role that these forms of memory play in support of cognition more broadly. Such efforts also promise to advance our understanding of how words, concepts, and meaning, as well as episodes and events, are instantiated and maintained in memory and will yield new insights into our two most quintessentially human abilities: memory and language.

## Introduction

Nearly 50 years ago, Tulving (1972) proposed that memory research may benefit from observing a distinction between episodic and semantic memory. In distinguishing episodic and semantic memory, Tulving stated that episodic memory referred to knowledge “*about temporally dated episodes or events, and temporal-spatial relations among these events*” and noted that such memory is stored “*in terms of its autobiographical reference to the already existing contents of the episodic memory store*” (Tulving, 1972, p. 385). Semantic memory was defined as the “*memory necessary for the use of language. It is a mental thesaurus, organized knowledge a person possesses about words and other verbal symbols, their meaning, and referents, about relations among them, and about the rules, formulas, and algorithms for the manipulation of these symbols, concepts, and relations*” (Tulving, 1972, p. 386). This distinction was offered by Tulving as something of a thought experiment, one that he proposed might have utility in understanding, and accounting for, the broader range of memory phenomena and experimental findings of the time. Indeed, Tulving stated, “*I will refer to both kinds of memory as two stores or as two systems, but I do this primarily for the convenience of communication, rather than as an expression of any profound belief about the structural or functional separation of the two. Nothing very much is lost at this stage of our deliberations if the reality of the separation lies solely in the experimenter’s and the theorist’s, and not the subject’s mind*” (Tulving, 1972, p. 384).

Despite Tulving’s own ambivalence, at least in his early writings, about the reality of the distinction between episodic and semantic memory, this distinction has persisted and has formed the foundation for decades of theoretical and experimental work in the cognitive neuroscience of memory. Considerable effort has been directed towards understanding the similar and unique features of episodic and semantic memory as part of a broader effort to characterize the neurobiology of memory, its functional divisions, and neuroanatomical correlates (e.g., Cohen and Squire, 1980; Squire, 1992; Tulving and Markowitsch, 1998; Thompson-Schill, 2003; Ryan et al., 2008; Greenberg and Verfaellie, 2010; Henke, 2010; Ranganath, 2010; Hannula and Duff, 2017). A key finding, and area of broad consensus, is that the hippocampus, and surrounding medial temporal lobe (MTL) structures, play a critical role in the encoding and subsequent retrieval of new long-term episodic memories (Cohen, 1984; Squire, 1992; Cohen and Eichenbaum, 1993; Gabrieli, 1998; Davachi, 2006; Eichenbaum et al., 2007; Rugg et al., 2015). A key source of evidence for the link between episodic memory and the hippocampus came from studies of patients with hippocampal damage who had profound deficits in acquiring new information about their daily lives and experiences (Scoville and Milner, 1957; Damasio et al., 1989; Corkin, 2002; Rosenbaum et al., 2005). This observed deficit was in contrast to the seemingly preserved ability of these patients to recount episodes from the remote past (or at least relative to events experienced since the onset of amnesia) and the ability to acquire new skills and habits (non-declarative, or procedural, memory).

But, what was the status of semantic memory? Was semantic memory, like episodic memory, also critically dependent on the hippocampus? And, given hippocampal damage, are deficits in episodic and semantic memory observed in tandem? This was a central question in the field. One prominent proposal was that semantic and episodic memory comprise, or depend upon, a unitary memory system, the declarative memory system, and that hippocampal damage would yield similar deficits (Cohen, 1984; Cohen and Eichenbaum, 1993; Squire and Zola, 1996; Cohen et al., 1997; Eichenbaum, 1998). An alternative proposal was that episodic and semantic memory formation was independent and could be acquired or damaged in isolation (Kinsbourne and Wood, 1975). Yet another proposal suggested that all memories start out as episodic and that over time some become semantic through processes of semantization or decontextualization (i.e., whereas episodic memories are bound to temporal and spatial contexts, the absence or loss of this specific context makes such memories semantic; for discussion and review, see Meeter and Murre, 2004).

In contrast to the clear and definitive evidence for the link between the hippocampus and episodic memory, the role of the hippocampus in semantic memory has been a topic of considerable debate. This debate stems from highly variable results from studies of new semantic memory learning in amnesia (as measured by different groups, in different patient populations, with different paradigms) ranging from reports of profound impairment to full preservation, and various degrees of deficit and ability in between. While interest in the (in)dependence of semantic memory and the hippocampus remained high, as evidenced by a number of reviews and commentaries (e.g., Mishkin et al., 1998; Squire and Zola, 1998; Manns et al., 2003; Manns, 2004; Moscovitch et al., 2006), research over the intervening decades did not produce sufficient data to form a core of consistent findings that could definitively adjudicate between competing views or resolve the debate.

More recently, a number of significant advances in the field have occurred, resulting in new provocative data on a robust role of the hippocampus in semantic memory. Thus, this is an ideal moment to revisit this debate, to re-evaluate the data and methods that informed traditional views on this topic, and to synthesize new findings. In line with these advances, our review has two primary goals. First, we provide a historical lens with which to evaluate, update, and contextualize the literature on semantic memory and the hippocampus. In doing so, we look back on this body of work and note a shift in the framing of the research questions, hypotheses, and levels of evidence that altered the trajectory of this line of research away from the original question on the extent to which semantic and episodic memory depends on the hippocampus in parallel and instead moved towards studies on new semantic learning in amnesia largely in isolation from episodic memory. While this “hypothesis drift” was likely unintentional, it seems to have gone unnoticed or at least not discussed in the literature. One consequence is that more recent researchers have inferred an answer to the original question (do episodic and semantic memory have shared dependence on the hippocampus?) based on evidence that was generated in response to the new reframed (drifted) question (can any new semantic learning be accomplished in amnesia?). We note that during this same time period, the number of investigations into the role of the hippocampus in episodic memory grew exponentially relative to those on semantic memory, based on powerful methods and techniques capable of measuring and quantifying episodic memory, and its perceptual, temporal, and spatial richness. Likewise, advances in theoretical proposals for understanding the nature and function of episodic memory have outpaced those related to semantic memory. We conclude that as time passed, researchers not only moved further away from the question originally posed about (in)dependence of episodic and semantic memory vis a vis the hippocampus but were also increasingly ill-equipped (methodologically and theoretically) to address it. The second goal of this review is to provide a critical reporting and synthesis of new findings on the role of the hippocampus in semantic memory. These advances have significant implications for understanding the role the hippocampus may play in the various stages of acquisition, maintenance, activation, and use of semantic memory in processing, paralleling what we have learned about the role of the hippocampus in the acquisition, maintenance, activation, and use of episodic memory.

We will argue here that the hippocampus is critical to both episodic and semantic memory. With the theoretical and empirical advances in the study of semantic memory and its neural bases, we can see that the depth and richness of semantic compare favorably to that of episodic memory and that they are both highly flexible, (re)constructive, relational and multimodal systems reliant upon the properties of the hippocampus. Such advances promise to illuminate our understanding of how words, concepts, and meaning are instantiated and maintained in memory, and then activated and used on-demand, just as well as, and in the same ways as are episodes and events.

Before we begin, we should acknowledge that our focus in this review is on semantic memory and that our approach is from the specific vantage point of the debate in the cognitive neuroscience literature on the extent to which semantic and episodic memory depends in tandem on the hippocampus. We place special emphasis on work with neurological patients as it has figured prominently in the history of this literature and it speaks to issues of necessity. Thus, our review does not cover semantic theory or its history (e.g., Grice, Locke, Searle) and we do not review the neuroimaging literature on semantic memory (although see these reviews: Martin and Chao, 2001; Thompson-Schill, 2003; Binder et al., 2009; Binder and Desai, 2011). Our review also places a special focus on the hippocampus. While the cortices of the MTL (e.g., perirhinal, parahippocampal, entorhinal) have been shown to contribute to episodic and semantic memory (e.g., Davachi et al., 2003; Davies et al., 2004; Eichenbaum et al., 2007; Clarke and Tyler, 2015), it is the shared, and often focal, hippocampal damage across patient studies that offer the most compelling evidence for the role of the hippocampus in semantic memory.

We start this review by reexamining and providing a critical context for the historical literature on the ability of individuals with hippocampal damage and resulting anterograde amnesia to acquire new semantic memory and on the integrity of their remote semantic memory, and show how it directly connects to current understanding of the role of the hippocampus in episodic memory.

## New Semantic Learning and Remote Semantic Memory in Amnesia

### New Semantic Learning in Amnesia

The neuropsychological and neuroanatomical description of the seminal case of HM provided significant insight into the organization and neural correlates of human memory (Scoville and Milner, 1957; Corkin, 2002). It also provided the early testing ground for the question of whether hippocampal damage produced commensurate deficits in episodic and semantic memory. The emphasis of this work was on new learning. Empirical testing and behavioral observation revealed that HM had a profound deficit in the encoding and subsequent retrieval of new episodic memory while his ability to recall and recount detailed events and experiences from his remote past appeared intact. It also appeared that HM’s remote semantic memory was intact. He did not present with aphasia, was able to name objects, hold conversations, and answer questions about remote facts and knowledge acquired long before the onset of his amnesia. The open question then was whether the deficit in acquiring new semantic memory mirrored his deficit in acquiring new episodic memory.

Before examining this literature, it is important to consider what the shared dependence of episodic memory and semantic memory on the hippocampus might look like. Because this review largely focuses on the abilities and deficits of patients with hippocampal amnesia, let’s consider various outcomes and standards for evaluating the data. One standard for confirming that episodic and semantic memory depend on the hippocampus in tandem might be to require equivalent levels of performance, ability, or deficit, in both episodic and semantic memory, in patients with amnesia. Another standard might be to require impairment in both systems but accept variable degrees of a deficit. In contrast, if the two systems are independent, then one might expect a dissociation, with impaired ability in one area and preserved ability in the other. Irrespective of the standard applied, addressing this question has proven difficult due to challenges in equating task demands and characteristics of to-be-learned stimuli across memory systems, and in quantifying lesion extent and residual abilities across patients with amnesia. Thus, a more common approach has been to examine the ability of patients with hippocampal amnesia to learn new semantic information and compare their performance to healthy comparison participants (to establish the existence of a deficit), and then to compare (often in relative rather than quantitative terms) the magnitude of these deficits across systems. Here, one standard might be to require that patients with hippocampal amnesia and healthy comparison participants perform similarly on all aspects of semantic learning (i.e., amount of information acquired, learning rate, generalization). Another standard might be to accept any level of patient learning even if it differs significantly from what healthy individuals are able to acquire, so long as this learning seems different or better than patients’ episodic memory ability. As, we will see below, each of these approaches has yielded variable levels of evidence and different groups have applied different standards that have shifted over time.

#### New Semantic Learning in Amnesia: None, or at Least Not Much

Gabrieli et al. (1988) were among the first to examine new semantic memory in HM. They tested the ability of HM and seven healthy comparison participants to acquire the meanings and synonyms of eight low-frequency words (e.g., *quotidian, manumit, hegira*) under formal laboratory conditions (i.e., each word presented individually with its definition, participants read each word and definition aloud). Knowledge was tested without asking for recall or recognition of any explicit, episodic aspect of prior experience with the words. Gabrieli et al. (1988) reported that HM did not learn any of the new words, or their synonyms, failing to ever reach criterion with experimental sessions aborted after 20 trials. In contrast, controls rapidly acquired the meanings of the new words and their synonyms, and were able to generalize these word meanings to new semantic contexts (e.g., in a sentence). While controls reached criterion in 7.3 trials, on average, it was estimated that HM would have required 335 trials to do so. That HM failed to learn the meaning of even a single word was taken as strong evidence for a profound impairment in semantic memory. The authors reported that “*HM could not learn, in a laboratory setting, the meaning of any word that he did not already know*” (Gabrieli et al., 1988, p. 161). The interpretation was that the impairment in new semantic learning was so severe, it seemed commensurate with that seen in the episodic domain; therefore, both episodic and semantic memory appeared to depend in common upon the hippocampus.

Future studies provided additional evidence for a deficit in learning new semantic information in HM (Postle and Corkin, 1998) and studies with other MTL patients provided more evidence that patients with amnesia were impaired on both semantic and episodic memory to a similar degree (Hamann and Squire, 1995). Hamann and Squire asked a group of amnesic patients to learn new facts (40 three-word sentences such as “MEDICINE cured HICCUP”) and tested their knowledge by presenting them with a sentence fragment to complete (e.g., MEDICINE cured ________). The amnesic patients learned at an abnormally slow rate (progressing from 0% to 19% correct vs. better than 75% for controls) and acquired a few exemplars relative to controls. Patient EP, a severely amnesic patient who is reported to have no detectable episodic memory, participated in this study. Like HM in the Gabrieli et al. (1988) study, EP exhibited no semantic learning at all. In recounting these data later, Squire and Zola (1998) commented that “*in a patient with no detectable capacity for episodic memory, there was also no detectable capacity for acquiring semantic knowledge*” (p. 208). Studies like these provided strong evidence for a deficit in new semantic learning in hippocampal amnesia, suggesting commensurate deficits in semantic and episodic memory and providing support for their shared dependence on the hippocampus for normal functioning. As, we will see below, however, the emphasis researchers (including ourselves) placed on *zero* semantic learning and *no detectable capacity* for semantic memory likely shifted the null hypothesis for subsequent studies.

#### New Semantic Learning in Amnesia: Some

Numerous groups have now shown that under some conditions, individuals with amnesia can acquire some new semantic memory. The majority of these studies used tasks and manipulations that attempted to promote new learning by reducing errors or interference (e.g., prevent incorrect information from interfering with recall of correct information; Glisky, 1992), and increasing the meaningfulness (e.g., embedding word lists in high-imagery narratives; Kovner et al., 1983) or semantic relatedness (e.g., table-chair; Shimamura and Squire, 1984) of the to-be-learned stimuli rather than traditional learning (study-test) methods. An approach popularized by Glisky et al. (1986) was to teach new semantic information to memory-impaired individuals using a technique called vanishing cues, a learning strategy under the umbrella approach of errorless learning. The general motivation for using errorless learning strategies to teach new information to individuals with memory impairment came from a growing body of work showing more success in approaches that compensate for specific memory problems compared to those aimed at restoring memory ability (Wilson and Moffat, 1983). Glisky et al. (1986) taught amnesic patients to associate computer terminology (e.g., save, run, boot) with their definitions. Consistent with the premise of reducing opportunities for patients to make errors, when patients could correctly produce the correct answer following a particular cue, they were then trained to respond to reduced cues (cues with letters removed). If participants made an error, letters were added to the cues until correct answers were remembered. In the Glisky et al.’s (1986) study, this technique was successful in teaching four patients with severe amnesia to learn some new computer vocabulary. Using similar learning strategies, patients with hippocampal amnesia can acquire some new semantic information (e.g., Tulving et al., 1991; Gordon Hayman et al., 1993; Baddeley and Wilson, 1994; Bayley and Squire, 2002; Skotko et al., 2004; Stark et al., 2005; Dewar et al., 2009; Hilverman et al., 2016). Across all of these studies, however, irrespective of method or technique, while the patients with amnesia do show some new learning, the learning is impaired and performance is far below what healthy participants can or would be expected to achieve. Patients with hippocampal amnesia acquire only a fraction of what controls learn, their rate of learning is abnormally slow [e.g., in Bayley and Squire (2002) a patient required 48 trials instead of the four trials required by controls], and, unless variability is built into the training procedure, the information they acquire is often rigid and inflexible (Stark et al., 2005).

Building on previous studies showing evidence for some new semantic learning in hippocampal amnesia, O’Kane et al. (2004) returned to HM, who is considered the gold standard case of amnesia as he was the first and most extensively studied case of amnesia in the literature. O’Kane et al. (2004) tested HM on his incidental learning of the names of individuals who had become famous after the onset of his amnesia using a 2-alternative forced-choice (AFC) recognition of famous names design and free recall of associated semantic information. They noted that, “*Until recently, it seemed unlikely that any semantic knowledge could be acquired following extensive bilateral damage to the MTLs*… and stated that “*whether the hippocampus proper is necessary for all semantic learning, or whether some degree of semantic learning can occur in the absence of a functioning hippocampus*” was an open question (O’Kane et al., 2004, p. 417). HM’s performance on the task was above zero indicating he had acquired new semantic memory since the onset of his amnesia. But, this learning was certainly not normal or in line with the performance of healthy participants. HM generated semantic knowledge about only a fraction of the famous people known to the comparison participants and what knowledge he had was sparse and highly variable and inconsistent, particularly relative to his knowledge of pre-morbidly acquired famous people (e.g., HM might correctly identify someone as famous but not know their sex). The conclusion was that, “*Although HM’s semantic learning was clearly impaired, the results provide robust, unambiguous evidence that some new semantic learning can be supported by structures beyond the hippocampus proper*” (O’Kane et al., 2004, p. 417).

The study by O’Kane et al. (2004) represents, and is reflective of, a significant turning point in the literature. Looking back on this literature, the findings of, and emphasis on, zero learning or floor level performance on tests of new semantic learning in amnesia by Gabrieli et al. (1988) and Hamann and Squire (1995) likely resulted in “hypothesis drift.” We borrow the term hypothesis drift from Nadel (1991) to reference the phenomena of recasting the hypothesis to accommodate new, often contradictory, data. We can see this drift represented in how O’Kane et al. (2004) framed the question for their study. Whereas the earlier studies were asking if episodic and semantic memory each had a dependence on the hippocampus, O’Kane et al. (2004) were asking a different question: Can any new semantic learning be accomplished in amnesia and can semantic learning occur independent of the hippocampus? This hypothesis drift was likely unintentional and went largely unnoticed, such that the bar for demonstrating new learning remained the same, despite the change in the research question. As a result of earlier studies with HM and EP, the bar for demonstrating “new learning” was set so low that any performance better than zero would be noteworthy.

Indeed, taken together with the growing body of studies documenting *some* new semantic learning in amnesia, HM’s “*clearly impaired*” learning was interpreted as a viable challenge to the notion of commensurate deficits in episodic and semantic memory in amnesia and as evidence for the independence of semantic memory from the hippocampus. Some authors even argued that the semantic learning observed in amnesia was “*partially or perhaps even wholly preserved*” although the experiments contained no control group or direct comparison to experimental episodic memory performance (Tulving et al., 1991, p. 614).

These studies reflect another, perhaps more subtle, drift in framing the hypothesis: that the hippocampus alone supports semantic memory. Returning to the original proposals on the shared dependence of episodic and semantic memory on the hippocampus, the hypothesis was never that dependence on the hippocampus was exclusive, just that it was necessary (e.g., Squire and Zola, 1996; Cohen et al., 1997; Baddeley et al., 2001).

In our view, given the similarities between semantic and episodic memory representations (e.g., both require relational binding of multimodal information, expressed flexibly in novel contexts), a shared dependence on the hippocampus across memory systems makes intuitive sense. Further, just as we have come to understand that the full capacity and expression of episodic memory depends critically on a network of brain structures, including but not limited to the hippocampus (e.g., Buckner and Carroll, 2007; Ritchey et al., 2015; Wang et al., 2015; Moscovitch et al., 2016), so too, semantic memory, in its full capacity, relies on a network that includes, but goes beyond the hippocampus (Binder et al., 2009; Binder and Desai, 2011). In fact, there is considerable neuroanatomical overlap in the semantic network and the default-mode network, which supports episodic memory (Binder and Desai, 2011; Irish et al., 2016; Renoult et al., 2019).

A common interpretation across studies of new semantic learning in amnesia was that, even if fully normal semantic learning could not be obtained in the presence of hippocampal damage, some degree of semantic learning could be supported by structures beyond the hippocampus, specifically those associated with the non-declarative memory system. The connection between the limited semantic memory ability in adults with amnesia and their preserved non-declarative memory ability fits well with the properties of the non-declarative memory system (e.g., slow, inflexible, experience-dependent; Reber et al., 1996). Furthermore, a role for non-declarative memory processes in semantic memory acquisition, in concert with hippocampal-dependent memory processes, also fits well with its proposed role in normal word learning ability in healthy individuals (Davis and Gaskell, 2009; Gupta, 2012). Viewed from the perspective that non-declarative memory processes are part of normal word learning, it becomes less surprising that such processes are used to support semantic learning in amnesia and more striking how impoverished and difficult new semantic learning is without the contribution of the hippocampus.

Acknowledging the hypothesis drift and reframing of the research questions that occurred in the literature, and its impact is important for several reasons. To our knowledge, there has been no explicit discussion of it in the literature. One consequence is that readers and researchers alike have inferred an answer to the original question (do episodic and semantic memory have shared dependence on the hippocampus?) based on evidence that was generated in response to the new reframed (drifted) question (can any new semantic learning be accomplished in amnesia?). As we will discuss in more detail below, this hypothesis drift likely changed the types of data, and levels of evidence, that have accumulated over the intervening decades. We propose this has had cascading effects on the direction the field has gone and the pace of theoretical and methodological development in the area of semantic memory.

#### New Semantic Learning: Normal, or at Least a Lot, but…

Several groups have now reported normal semantic memory in the context of severe deficits in episodic memory (e.g., Sharon et al., 2011; but, see Warren and Duff, 2014, 2019; Elward et al., 2019). The work on semantic learning in patients with developmental amnesia by Vargha-Khadem et al. (1997) is the most highly cited on this topic and is considered the most compelling evidence for the dissociation in new learning of episodic and semantic memory in the literature (Vargha-Khadem et al., 1997). They reported on three cases of developmental amnesia, individuals who sustained selective hippocampal damage early in life; at birth for one case, and at ages 4 and 9 in the other two cases. At the time of the report, these three individuals were in their teens and early twenties. Neuroimaging assessment revealed hippocampal volumes between 43% and 61% of the mean values of a healthy comparison group but showed surrounding MTL cortices to be unaffected. It is important to note that while the neuroimaging assessment indicates that there is still residual hippocampal tissue present, it has been suggested that a reduction in hippocampal volume of approximately 40% likely represents a near-complete loss of hippocampal neurons (Gold and Squire, 2005). Neuropsychological data showed severe deficits in episodic memory across a battery of tests (e.g., the logical memory and visual memory subtests of the Wechsler Memory Scales (WMS), Children’s Auditory Verbal Learning Test (AVLT), Rey-Osterreith Complex Figures Test). The participants also displayed significant difficulty with episodic memory in their day-to-day lives. Yet, despite these severe episodic memory deficits, these three individuals acquired language, semantic knowledge and factual information that placed them in the low-average to average range on standardized assessments, and were able to attend mainstream school. The authors concluded that developmental amnesia “*produces a severe loss of episodic memory but leaves general cognitive development, based mainly on semantic memory functions, relatively intact*” (Vargha-Khadem et al., 1997, p. 376). Furthermore, given the level of semantic learning achieved in the context of significant episodic memory deficits and hippocampal pathology, the authors argued that normal levels of semantic learning can be achieved independent of the hippocampus. These data were remarkable on many levels. Prior to this publication, the prediction was that early hippocampal pathology would produce widespread and devastating cognitive and intellectual deficits. The amount of semantic learning acquired in these cases far exceeded what was predicted. Furthermore, the level of semantic memory acquired in developmental amnesia seemed strikingly superior to that achieved in adult cases.

There are well-acknowledged challenges in comparing data from developmental and adult-onset populations (Squire and Zola, 1998; Elward and Vargha-Khadem, 2018). One critique of the developmental amnesia work has been that semantic memory was not tested as directly, or formally, in laboratory settings, as was episodic memory, in contrast, for example, in the way it was tested in patient HM (Gabrieli et al., 1988). This makes it difficult to compare quantitative measures of performance on standardized tests of episodic memory (where individuals encode and recall newly acquired information in the same testing session) with extensive, repeated real-world exposure to semantic memory across time and naturalistic contexts. However, note that standardized episodic and standardized semantic memory tests are not well equated either. Episodic tests (e.g., AVLT, WMS) examine what an individual acquires in the testing session and semantic tests (picture vocabulary tests like the Boston Naming Test or Pyramids and Palm Trees Test) examine vocabulary and semantic knowledge acquired and reinforced over a lifetime.

There are now more formal, laboratory studies of new semantic learning in cases of developmental amnesia in the literature (Elward and Vargha-Khadem, 2018). When examined using laboratory tasks that more closely mirror those used in the adult-onset literature, the pattern of deficit in developmental amnesia seems remarkably similar to the adult-onset cases: the learning rate is slower (Gardiner et al., 2008; Elward and Vargha-Khadem, 2018), less information is acquired (Baddeley et al., 2001) and there is less evidence of generalization relative to controls. The learning deficit is most striking in tasks that require rapid learning and free recall, supporting the notion that the hippocampus is critical for rapid and efficient semantic learning, whereas performance is significantly better, or even similar to controls, when additional learning trials are provided and when learning is measured with recognition or cued recall (Elward and Vargha-Khadem, 2018). Additional evidence for a semantic memory deficit in developmental amnesia comes from Blumenthal et al. (2017) who asked a patient to generate semantic features for object concepts. They reported abnormal patterns of feature generation and typicality ratings in the patient with developmental amnesia relative to controls. The authors attributed these semantic memory deficits to impairments in hippocampal binding mechanisms and suggested that the dissociation between semantic and episodic memory in developmental amnesia may not be as complete as previously conceptualized (Blumenthal et al., 2017).

Duff et al. (2006) have also reported an intact rate of learning for semantic information in adults with hippocampal amnesia. In their study, four patients with hippocampal amnesia completed a referential communication task with a familiar partner (spouse, friend). The patients sat across from their partner and each had a board with 12 numbered spaces and a set of 12 cards displaying Chinese tangrams (i.e., abstract black and white figures with no established names but which could be perceived as people, animals, or objects). A low barrier was between them preventing a view of each others’ cards but allowing them to see each other’s facial expressions and gestures. The patients with amnesia were the directors and communicated to their partner (always the matcher) how to complete the board with the cards so that at the end of a trial the two boards looked alike (i.e., their cards were in the same numbered spaces on each board). The task was presented as a game and pairs were instructed to communicate freely and have fun. Despite severe episodic memory impairments, the amnesic participants developed and used unique labels for the cards. Across trials, these labels became increasingly concise and simplified. Most strikingly, the rate of learning exhibited by amnesic participants, measured by the reduction in time and words necessary to complete each trial, did not differ from that of healthy participants. The long-term retention of this new learning at 30 min, 6 months, and even 2 years for one participant did not differ between groups. These results were the first to show an intact rate of new semantic learning in adult-onset amnesia in a social-communicative learning paradigm. The results also have significant implications for rehabilitation and highlight the role of social interaction as a means of facilitating new learning in individuals with memory impairment.

Yet, there is a caveat: the learning did not require the acquisition of new arbitrary relations, an ability that relies critically on the hippocampus and that is part of what normal semantic learning typically demands. The patients with amnesia negotiated meaningful labels for the tangrams using pre-existing semantic information (e.g., “siesta man” for a figure that could be viewed as a person lying against a tree). When patients with hippocampal amnesia are the matchers, and their partners are the directors (i.e., the ones generating the perceptual and linguistic perspectives), the patients show little learning, likely because the to-be-learned labels generated by their partners are, in the minds of the patients, arbitrarily related to the tangram figures (Gupta Gordon et al., 2018). Thus, patients with hippocampal amnesia can be successful at learning new semantic information when the task does not demand hippocampal mediated learning (e.g., arbitrary relational binding) and, in the context of real-world social communication, this learning can even be achieved at a normal rate. The role of social interaction and communication in new semantic learning warrants further consideration. Not only is social interaction the canonical context for semantic learning in development and language acquisition, but it is also the context for the most impressive examples of new semantic learning in amnesia, even if not fully normal, whether in developmental or adult-onset cases of amnesia (Koutstaal, 2019). This is particularly true for individuals with developmental amnesia who have learned a wealth of semantic information outside the laboratory.

Looking back on all the evidence of new semantic learning in amnesia, there is yet to be a replicable example of fully normal semantic learning (i.e., where the rate and amount of learning between amnesic patients and controls are similar and where the to-be-learned information encompasses the full range of demands (arbitrary binding) that are inherent to semantic memory). While there are learning conditions and formats that promote new learning in amnesia (e.g., errorless learning), when evaluated together and with a fixed standard, the empirical evidence shows that patients with dense amnesia following hippocampal damage fail to show normal acquisition of new semantic information, and thus supports the conclusion that the hippocampus plays a necessary role in the acquisition of new semantic memory. Taken altogether, although over time semantic and episodic memory have largely been studied separately, and increasingly apart from the early question of whether both forms of memory share a common neural substrate, the evidence is compelling that new semantic learning, like new episodic learning, relies critically on the hippocampus.

### Remote Semantic Memory in Amnesia

There has been an overwhelming consensus that remote semantic knowledge, acquired long before the onset of hippocampal pathology, becomes independent of the hippocampus *via* neocortical consolidation (McClelland et al., 1995) and is intact in amnesia. This view has been supported by data from patients with hippocampal amnesia on tests of linguistic knowledge: patients with amnesia do not have aphasia or semantic dementia, and they perform within normal limits on neuropsychological measures of vocabulary knowledge and naming (Kensinger et al., 2001). Further, patients with amnesia perform similarly to healthy participants on measures thought to assess remote word knowledge, like naming or matching a label with a phrase, definition, or sentence (Gabrieli et al., 1988; Verfaellie et al., 2000; Manns et al., 2003). Together, these data have been taken as evidence that patients with amnesia have intact remote semantic memory.

Perhaps the methods used in these studies are not fine-grained enough to detect impairment in patients with amnesia. Many of the tasks used in these studies were originally designed to detect aphasia or semantic dementia. As such, they capture differences in naming or linguistic ability at a coarse level. Examples of the procedures used include showing participants a picture of a common object, like an apple, and prompting patients to name it; matching the label *apple* to a definition like, *a sweet, red fruit*; and determining whether A-P-P-L-E is a real English word. While tests such as these are certainly useful in identifying a deficit in people with severe semantic or naming impairment, they do not capture more subtle deficits that may be evident in the remote semantic memory of patients with amnesia.

The same can be said of clinical tools commonly used for detection of deficits in people with semantic dementia or Alzheimer’s disease. Two such tools are the Semantic Memory Test Battery and the Boston Naming Test. These tests tend to be implemented with relatively few naming trials. When these tests are used in people with semantic dementia, naming impairment is evident. For example, studies with this population using just 28 items (Lambon Ralph et al., 2007) and 48 items (Schmolck et al., 2002) found deficits in naming. When these tests are used in patients with hippocampal amnesia, no naming impairment is found. Kensinger et al. (2001) tested patient HM using the Boston Naming Test—which included 42 black-and-white line drawings—and developed two picture naming tasks. One task had 96 colored pictures of objects and the other had 105 black and white drawings. HM performed similarly to controls on these tasks, leading to the interpretation that his remote semantic knowledge was intact.

More recently, researchers have sought to examine remote semantic memory in patients with amnesia using more sensitive measures that align more closely with approaches to study semantic richness (see below). Klooster and Duff (2015) examined how much information is associated with highly familiar words that were previously acquired in patients with amnesia and healthy and brain-damaged comparison participants. The tasks included a word associates test (identifying synonyms and common collocates), a word senses task (name all the senses of a word; e.g., lemon can be a fruit, a color, a defective automobile) and a word features task (name all of the features of a word; e.g., lemon tastes sour, is native to Asia, used in tea). Patients with amnesia performed significantly worse than healthy and brain-damaged comparison groups (i.e., patients with ventromedial prefrontal cortex damage), on all three measures of word knowledge. For example, patients with amnesia generated, on average, only half the number of features for common words (e.g., shirt) as comparison participants. The deficit in remote semantic memory was even evident on tasks where all the information was in view of the participants. For example, when provided with a word (e.g., sudden) and asked to endorse possible synonyms (e.g., beautiful, quick, surprising, thirsty), all of which were written on paper in view of the participants, individuals with amnesia were significantly less likely to identify the correct responses. Furthermore, this deficit was evident despite showing no differences from comparison participants on self-reported rates of familiarity (scoring familiarity on a 9-point scale) of words used in the word features and senses tasks. Importantly, the fact that the patients knew these words (i.e., had high familiarity ratings), suggests that they likely would have performed like comparison participants with traditional measures (e.g., naming) that only assess surface level semantic knowledge. Using tasks and measures that assess semantic richness, or depth of semantic knowledge, patients with hippocampal amnesia perform significantly worse than comparison groups suggesting impoverished remote semantic memory. These findings also raise the possibility that the hippocampus plays a long-term role in maintaining semantic representations across the lifetime.

Returning to studies of naming, deficits in remote semantic knowledge in amnesia are also evident when a more extensive set of items are probed. Dawood et al. (2018) conducted a naming task similar to previous studies in which patients with amnesia and comparison participants viewed color photographs of items and were instructed to provide a name for the picture. Unlike previous naming studies that all contained fewer than 100 images, this study used 1,458 items from the Bank of Standardized Stimuli (BOSS) database (Brodeur et al., 2010, 2014) that varied across a range of word features such as imageability, frequency, and familiarity. By using a wide range of image-word pairs, even subtle differences between patients with amnesia and comparisons in naming may be detected. Unlike previous tests of naming in this population, Dawood et al. (2018) found that patients with amnesia were less likely than comparison participants to correctly name the objects that they viewed. Furthermore, patients with amnesia were more likely to provide a general label for an object (e.g., *bird* for a *cardinal*) than healthy participants. Using a wider range of materials and a detailed analysis of error type provides further evidence of the impoverishment of remote semantic memory in amnesia.

Closer examination of language production also reveals group differences where patients with amnesia use words rated as less semantically rich relative to controls. Hilverman et al. (2017) analyzed the features of words used when patients with amnesia and healthy participants described events, both past and imagined. Features of words reflect characteristics of what the word describes (e.g., a word’s imageability measures the degree to which the word invokes an image in one’s mind). Although patients with amnesia are known to produce significantly fewer episodic details in their descriptions of events (Race et al., 2011), the specific words that are used are not necessarily related to the number of episodic details; similar representations can be communicated with the same amount of episodic details but using words that vary considerably in their imageability and concreteness. For example, one could say, “I was on a jetski on a nice summer day and water was hitting my face as I went across the lake” or “I was riding a jet ski on a bright summer day and water was spraying my face as I sped across the lake”. In both cases, the number of episodic details is the same, but the imageability and concreteness of the words used are much greater in the second account. Hilverman et al. (2017) found that patients with hippocampal amnesia used words that were significantly less imageable than healthy comparison participants. This was found even when controlling for number of overall features in the narrative and word frequency. This finding fits with data from Heyworth and Squire (2019) who found that in narrative recollections of a guided walk, patients with amnesia used higher-frequency and less concrete words than controls. Thus, even in semi-naturalistic speaking contexts, patients with amnesia demonstrate language use that is semantically impoverished.

These deficits in remote semantic memory are not present only in fine-grained aspects of language. Similar findings have been demonstrated in patients with amnesia when describing semantic knowledge acquired long before the onset of their amnesia. When prompted to recount fairy tales and bible stories, patients with amnesia produce fewer details than controls (Rosenbaum et al., 2009; Verfaellie et al., 2014). Patients with MTL lesions also show impairment in the general details and in the ordering of the main steps (Verfaellie et al., 2014). Further, a review of neuropsychological research on autobiographical knowledge demonstrated that patients with MTL damage were impaired on measures of autobiographical fact knowledge—a type of personal semantic memory—relative to comparison participants (Grilli and Verfaellie, 2014). Finally, patients with MTL damage are impaired relative to healthy participants at generating hypothetical meanings for novel word compounds (e.g., cactus carpet) suggesting that the hippocampus plays a role in relational and combinatorial semantic processing even when remote knowledge of the individual words appeared intact (Keane et al., 2019).

There is growing evidence of remote semantic memory impairment in amnesia. These impairments may mirror deficits in remote episodic memory in amnesia. Close examination of remote episodic memory in amnesia reveals a lack of specificity, detail, and richness relative to healthy participants (e.g., Rosenbaum et al., 2008; St-Laurent et al., 2014; Robin et al., 2019) and support the proposal that the hippocampus plays a long-term or permanent role in the maintenance of episodic memory representations (Nadel and Moscovitch, 1997). To test the notion than hippocampus plays a long-term or permanent role in the maintenance of both episodic and semantic memory, researchers will need to develop/apply methodological approaches to the study of semantic memory that mirror those used to study episodic memory in terms of their ability to capture the breadth and richness of the multimodal and relational features that are inherent to both forms of memory.

## Methodological and Theoretical Approaches to Studying Episodic and Semantic Memory

One challenge of testing the shared dependence of episodic and semantic memory on the hippocampus has been equating task demands and characteristics of the to-be-learned stimuli across memory systems. A consequence of the early confirmation and consensus on the role of the hippocampus in episodic memory (while the early data on semantic memory were more equivocal) is that the number of investigations and highly sophisticated experimental designs to study episodic memory have significantly outpaced those on semantic memory. Consistent with proposals that view the hippocampus as playing a critical role in relational binding and in the flexible (re)construction and (re)combination of rich multimodal features of events and experiences (Eichenbaum and Cohen, 2001; Schacter and Addis, 2007; Ranganath, 2010; Yonelinas, 2013; Rubin et al., 2017), the field now has a diverse set of methods for capturing and quantifying the relational features and contextual richness of episodic memory. For example, to study episodic memory, we have coding schemes for rating and quantifying the spatial, temporal, and perceptual vividness and richness of event narratives (e.g., Levine et al., 2002), experimental designs for examining how episodes are (re)constructed, (re)combined, and integrated across time, space, and people (e.g., Zacks and Swallow, 2007; Schacter et al., 2008; Schlichting and Preston, 2015; Eichenbaum, 2017) from photographs, text, and movie clips (e.g., Staresina and Davachi, 2009; Zacks et al., 2009; St-Laurent et al., 2014), and techniques like eye-tracking (e.g., Ryan et al., 2000) and entropy analyses (e.g., Lucas et al., 2019) that allow us to study episodic encoding and recall, and its organization, without asking participants to explicitly study or remember. In contrast, particularly in patient studies, the study of semantic memory still largely involves asking individuals to label pictures of famous faces and to learn facts or word-meaning pairings (Manns et al., 2003; Sharon et al., 2011). Our methods and techniques for measuring episodic and semantic memory, and equating task demands and stimuli, are further apart than they were decades ago.

This lack of methodological depth and breadth in the study of semantic memory (and therefore the lack of substantive data) has made it difficult for researchers to offer complete and comprehensive theories across distinct forms of memory. For example, Nadel and Moscovitch (1997) note in their seminal paper laying out the points of similarity and divergence between standard consolidation models and their multiple trace theory that most studies of remote general semantic knowledge do not include detailed tests sensitive enough to detect deficits, which limits the comparison to other forms of memory. More recently, Yonelinas et al. (2019) proposed an alternative to standard systems consolidation theory called contextual binding theory which focuses nearly exclusively on the role of the hippocampus in episodic memory. Discussion of semantic memory was cursory, with the authors simply stating that whether or not contextual binding theory might be applied to semantic memory is an open question. Indeed, given the dearth of semantic memory studies with sufficient depth and sensitivity, this is all that can be said. This lack of data and methods may also make it more attractive, or tractable, to test hypotheses for which there are more established data and tools (as is the case in the area of episodic memory). Thus, over the past several decades, not only have researchers moved further away from testing if episodic and semantic memory has shared neural correlates, but, as a field, we are ill-equipped (methodologically and theoretically) to do so.

Other disciplines (e.g., psycholinguistics, semiotics, cognitive science) however, have conceptualized semantic memory as a knowledge system that is as rich, relational, and multifaceted as we have come to view episodic memory. From these fields come a set of tools and methods with increased sensitivity to capture a wider breadth of semantic memory phenomena than used in the memory literature to date. These methods may also have utility in attempts to equate task demands and stimuli across memory systems. In the next section, we review some of these broader approaches to demonstrate the similarities between episodic and semantic memory and to highlight their application to recent studies of hippocampal contributions to semantic memory.

## Semantic Memory as a Flexible, Constructive, Relational, and Multimodal System

Episodic memory is often described as a dynamic system capable of reconstructive and combinational processes that allow us to recollect about our past and simulate future events (Buckner and Carroll, 2007; Schacter and Addis, 2007). While the study of semantic memory in amnesia has often been reduced to word-definition pairs or recognition of famous faces or facts, other perspectives view semantic memory as a highly flexible, (re)constructive, relational and multimodal system that we use to create, represent, and extract meaning as we navigate our most fundamental interactions with the environment and each other (Rogers et al., 2004; Reilly et al., 2016). Like episodic memory, semantic knowledge is not a static repository of information. Rather, it grows and changes as we continuously acquire, integrate, and reinforce rich representations of the relations between words, their referents, and their relations with associated referents (Zettersten et al., 2018; Klooster et al., 2019). Indeed, it is estimated that the average English-speaking adult has acquired 12.5 million bits of information, the majority of which is lexical-semantic knowledge (Mollica and Piantadosi, 2019). These millions of bits of information are not isolated, but rather are interconnected and combined in both familiar and novel ways to represent and act in the world.

The acquisition of richly interleaved semantic knowledge is facilitated by the dynamic contexts in which words are learned and used. For example, single words are seldom learned or presented in isolation. Rather, words appear in rich contexts in which related words and concepts are also present, facilitating the development of interrelated semantic representations that can be flexibly deployed (Wojcik and Saffran, 2013, 2015; Wojcik, 2018). In addition to representing the relations between words and their referents, while adding increasing layers of nuance to the meanings of words over time (Ellis and Ogden, 2017), learners also represent relationships *among* lexical items, based on their co-occurrence in the ambient language (Arnon and Christiansen, 2017). That is, many sequences of words repeatedly co-occur in language and we encode those relations in addition to our knowledge of individual words (Pawley and Syder, 1980).

Like episodic memory, which is often characterized, and measured, in terms of its richness (e.g., episodic richness is the amount of multimodal information that is associated with a given event or experience; Levine et al., 2002; St-Laurent et al., 2014), semantic memory is also characterized, and measured, by richness. Semantic richness refers to the amount of information contained within or associated with a word or concept and it influences the speed and accuracy of behavioral responses (e.g., greater semantic richness is associated with faster and more accurate naming, lexical decision, categorization; Pexman et al., 2002, 2003; Duñabeitia et al., 2008; Grondin et al., 2009). Words and concepts that are richer, or associated with more information, are also better remembered (Hargreaves et al., 2012).

Semantic richness can be indexed or measured in a number of ways. It can be a metric of how many concepts, words, or features are associated with a specific word. Words with denser semantic neighborhoods—or words that are associated with many different words or concepts—are processed more quickly in naming, lexical retrieval, and lexical decision tasks (e.g., it is easier to retrieve the word “nurse” after viewing the word “doctor” than it would be having just viewed the word “grass;” Hargreaves and Pexman, 2012; Yap et al., 2012; Taler et al., 2013). Semantic richness can also be represented by how many sensory and perceptual features are associated with a particular word or concept. Indeed, words that are higher in imageability (can readily generate a mental image) and concreteness (can be imagined with the senses) are typically processed more quickly; it is easier to retrieve the word “banana”—something that can be seen, touched and tasted—than it is to retrieve the word “government”—a concept that is more abstract (e.g., Bennett et al., 2011). Semantic richness can also be a reflection of how many contexts a word or concept is associated with or can be successfully used in, typically measured across print sources (Adelman et al., 2006) but may also extend to distinct physical settings and speakers. Words that appear across more diverse contexts facilitate faster word naming and lexical decision times than do words that are just more frequently occurring. From the perspective of richness, there are obvious parallels between semantic and episodic memory. Manipulating semantic richness may be one way to help equate stimuli and task demands across memory systems. For example, work by Klooster and Duff (2015) and Hilverman et al. (2017) documenting deficits in semantic richness (e.g., the amount of information associated with a word) in patients with hippocampal damage highlights the shared role of the hippocampus in both episodic and semantic richness. Manipulating context as a form of semantic richness may also provide an opportunity to expand on, or test, existing memory theory. For example, contextual diversity is an interesting measure as it seems to capture the interaction of semantic representation and episodic experience rather than the extraction or decontextualization of semantics from episodes (e.g., semantization).

Rich semantic representations allow us to go beyond the literal meanings of words themselves, combining and integrating across concepts to communicate meanings that might otherwise be inexpressible (Katz, 1989). For example, the use of metaphor in human communication and thought is widespread (Lakoff and Johnson, 1980). To generate and comprehend metaphors (e.g., “my job is a jail”), language users create or identify *relations* between the metaphor topic (“job”) and vehicle (“jail”). Metaphor comprehension requires rapid processing of novel relations between seemingly disparate lexical items, and may, therefore, place high demands on the MTL relational memory system. Use of a metaphor is also inherently creative. Metaphors are thought to be a primary device driving lexical innovation (McGlone et al., 1994; Makkai et al., 1995). Metaphors help to fill lexical “gaps” in a language by extending existing words to describe novel categories and concepts. Another example is a conceptual combination. Speakers leverage the relations among lexical items to create new concepts and meaning by combining words and concepts from pre-existing knowledge stores (e.g., elephant-ferry; these words can be processed individually or as an integrated concept, an elephant ferry; Coutanche et al., in press; Lucas et al., 2017).

Metaphor and conceptual combination would seem to require the same compositionality and representational flexibility inherent in characterizations of episodic memory. That is, relational representations (semantic and episodic) can be broken down into constituent elements, which can then be combined and recombined in novel ways (Cohen and Eichenbaum, 1993; Cohen et al., 1997). Metaphor generation and conceptual combination clearly involve the combination of far-reaching mental representations and results in the generation of a verbal expression that creatively combines disparate concepts to provide the listener with novel insight. These creative combinatorial and constructive features of semantic memory processing and use are highly reminiscent of the flexible and creative (re)construction and (re)combination of episodic memory representations for remembering the past and imagining the future (Buckner and Carroll, 2007; Schacter and Addis, 2007). Indeed, individuals with hippocampal pathology are impaired in creative uses of language (Duff et al., 2009) including metaphor comprehension (Covington et al., 2017). Furthermore, work by Keane et al. (2019) on generating novel meanings for word combinations (e.g., cactus carpet) highlights the shared role of the hippocampus in both relational episodic processing and relational semantic processing.

Viewed through a broader interdisciplinary lens, episodic and semantic memory have many shared features including the depth and breadth of multimodal relational information they encompass and the constructive and flexible nature of their expression and use across contexts. While these shared features align closely with the processing capabilities of the hippocampus (e.g., relational binding, representational flexibility, compositionality; Cohen et al., 1997; Eichenbaum and Cohen, 2001), in the core memory literature, these broader semantic paradigms, and their (in)dependence to the hippocampal memory system, have, until recently been understudied. We next review recent developments in our understanding of the hippocampus that further align, and demonstrate, the capacity of the hippocampus to meet the processing demands of semantic memory use and processing.

## Extending the Reach of the Hippocampus and Its Role in Semantic Memory Processing

The hippocampus has long been associated with long-term memory. Converging evidence has challenged the traditional view that the hippocampus exclusively supports long-term memory, showing that the hippocampus plays a critical role in memory for relations over very short delays, and even when there are no delays at all, on the timescale of short-term or working memory (Hannula et al., 2006, 2017; Olson et al., 2006; Hannula and Ranganath, 2008). These findings suggest that new hippocampus-dependent representations are available rapidly enough to influence ongoing processing when: new information is perceived; old information is retrieved; and representations are held on-line to be evaluated, manipulated, integrated, and used in service of behavioral performance. That is, the hippocampus is critical not only for the ability to form new enduring memories and to recover the past, but also for the creation, maintenance, updating, and use of on-line representations in support of ongoing information processing. These findings raise the possibility of hippocampal involvement in real-time semantic processing.

The hippocampus has also long been associated with explicit and conscious processing. Recent work, however, implicates the hippocampus in the incremental and implicit/unconscious processing of arbitrary relations (for review, see Hannula and Greene, 2012), suggesting that consciousness alone is not a reliable predictor of what neural region or memory system contributes to a given behavioral phenomena. Although implicit semantic processing tasks have often been assumed to be hippocampal independent, these new findings raise the possibility that the hippocampus may contribute to some aspects of unconscious or implicit semantic processing (also see Gaskell et al., 2019). Initial support for such a prediction comes from data pointing to hippocampal contributions to statistical learning, the process by which individuals uncover patterns in their environment by tracking co-occurrence frequencies amongst stimuli. In language, statistical learning is the proposed mechanism by which we learn to segment words from continuous speech (Saffran et al., 1996), uncover grammatical structure (Gómez, 2002; Saffran and Wilson, 2003), and learn to recognize the phonotactic, orthographic, and morphological regularities (Chambers et al., 2003; Pacton et al., 2005). There is also evidence to suggest that statistical learning mechanisms contribute to semantic knowledge by supporting the mapping of word meanings onto word forms (Graf Estes et al., 2007; Lany and Saffran, 2011; Lany, 2014). Although considered an implicit learning process, recent work (imaging and patient studies) demonstrates a role for the hippocampus in the tracking of statistical regularities in the environment, across stimulus modalities (Schapiro et al., 2012, 2014; Covington et al., 2018).

Taken together with the long-acknowledged role of the hippocampus in relational binding, these new findings have significant implications for understanding the role the hippocampus may play in various stages of acquisition, maintenance, activation, and use of semantic information. By combining broader theoretical and methodological approaches to semantic memory and the functionality of the hippocampus, there is a growing literature demonstrating hippocampal contributions to semantic progressing in the moment. Next, we highlight studies that have documented hippocampus contributions in on-line semantic memory processing.

### Hippocampal Contributions to Semantic Processing in the Moment

A particularly innovative approach to studying hippocampal contributions to on-line semantic memory processing comes from intracranial recordings from depth electrodes in patients with intractable epilepsy. These studies have the advantage of a high degree of both spatial and temporal specificity, allowing for tests of the nature and time course of hippocampal contributions to semantic processing. Two such studies demonstrate hippocampal coding for semantic representations depending on a similar mechanism to hippocampal coding for space/episodes: hippocampal theta power. The role of the hippocampus is well-established in the encoding of relations for representing and navigating physical space (O’Keefe and Nadel, 1978; Nadel, 1991). Solomon et al. (2019) ask if hippocampal theta oscillations represent semantic distances between words (i.e., the similarity or likeness in meaning between words as measured by corpus analysis), similar to how these same oscillations code for relations in physical space. In this study, patients with depth electrodes with contacts on hippocampus completed study and recall of sets of 12-item lists. During recall, patients demonstrated the expected behavioral pattern of clustering list items based on both their temporal relations (e.g., words in close serial proximity during the study were recalled in clusters during recall) and also based on semantic relations (e.g., words closer in semantic space were recalled in clusters during recall). Hippocampal theta power prior to the retrieval event was predictive of the semantic relationship in the two subsequently recalled words, suggesting that hippocampal theta power codes for semantic relatedness in multi-dimensional word space. These data are striking as they suggest a role for the hippocampus in tracking and representing the relations among words in semantic memory in a manner that is similar to how the hippocampus tracks and represents relations in physical space and events in episodic memory.

Piai et al. (2016) demonstrated relationships between hippocampal theta power and semantic processing during language comprehension. In contrast to the list learning in the Solomon study, patients in the Piai study were not required to learn any new information. In this study, the patients listened to sentences with the final word omitted and were then presented with a picture to name that could complete the sentence. In the experiment, half of the sentences presented to the patients began with a sentence stem that linguistically constrained the possible final word [e.g., “She locked the door with the” (picture: key)] while the other half were linguistically unconstrained [e.g., “She walked in here with the” (picture: key)]. The results demonstrated that constraining sentence stems facilitated the picture naming response, and that hippocampal theta power increased during the sentence stem for the constrained vs. unconstrained sentence stems, prior to the picture onset. Further analysis of these data demonstrated that the increases in theta power were related to increasing semantic associations between words in the sentence. Using latent semantic analysis (LSA), Piai et al. (2016) determined the “context-defining word” for each sentence (i.e., the word with the strongest LSA association to the final picture name). In the constrained condition, all patients demonstrated increased theta power at this keyword compared to the preceding word, a pattern that was not present in the unconstrained condition. These results demonstrated that the hippocampus contributes to tracking and building semantic associations across words, and suggest a role for the hippocampus in predictive language processing (also see Bonhage et al., 2015), consistent with its role in predictive processing in other domains (Buckner, 2010; Covington and Duff, 2016).

In a similar study to Piai et al. (2016), Jafarpour et al. (2017) examined patterns of hippocampal activity, specifically hippocampal high-frequency band (HFB) power, during the 0.5-second pause between the sentence stem and the appearance of the to-be-named picture. Greater HFB power was observed during the pre-picture period during the highly constraining vs. low constraint sentences, suggesting pre-activation of the expected semantic representation. Indeed, patterns of HFB power in the pre-picture and picture intervals were compared using time series analyses, and the degree of similarity between these patterns was higher for highly constrained items. These patterns of hippocampal HFB power were then compared to one another based on semantic similarity (as calculated using LSA). Results indicated that HFB power pre-activation patterns were more similar for pictures that were closer in semantic distance to one another.

Finally, data from intracranial recordings also suggest that the hippocampus contributes to word retrieval during picture naming (Hamamé et al., 2014). During picture naming, left hippocampal HFB power increased during the period between picture presentation and word production, relative to the pre-stimulus baseline. Peak-latency of this hippocampal response was predictive of participants’ trial-by-trial naming latency. The authors suggest that these results point to a role for the hippocampus in retrieving the arbitrary associations between objects and their names.

The results from these intracranial recording studies suggest that, in addition to the role for the hippocampus in the acquisition of new semantic memory and maintenance of remote semantic memory, the hippocampus also encodes, tracks, and builds semantic relations of previously acquired words during on-line sentence processing to create meaning in the moment and to facilitate communication (see Cross et al., 2018; Gaskell et al., 2019). The role of the hippocampus in semantic memory processing appears remarkably similar to the role the hippocampus plays in its support of episodic memory. Building on this work, interdisciplinary approaches to the study of hippocampal contributions to semantic memory promise to expand and refine the theories and methods across fields and may offer researchers new paradigms that will allow for integrating the study of episodic and semantic memory.

## Conclusion

It has been nearly 50 years since Tulving (1972) suggested that memory research may benefit from observing a distinction between episodic and semantic memory. Unquestionably, Tulving’s thought experiment has been a significant catalyst in the empirical and theoretical study of multiple memory systems. The shared neural correlates and the commonalities in processing and representation of semantic and episodic memory suggest to us that these forms of memory have more in common than Tulving’s initial distinction, and the work that followed, suggested (also see Renoult et al., 2019). Indeed, like episodic memory, semantic memory is a highly flexible, (re)constructive, relational and multimodal knowledge system. Furthermore, like episodic memory, semantic memory also depends critically on the hippocampus; patients with dense amnesia following hippocampal damage cannot acquire new semantic memory fully normally, just as they do not have the normal capacity for acquiring new episodic memory. This review highlights the role the hippocampus plays across nearly all stages of semantic memory including acquisition, maintenance, and processing in real-time.

There is growing recognition that the history of studying memory systems in isolation and the search for dissociations has led many to overlook the well-documented interdependence of episodic and semantic memory (Greenberg and Verfaellie, 2010; Ferreira et al., 2019; Renoult et al., 2019). Recent work also highlights the pivotal role semantic memory plays across many, if not all, forms of episodic memory, irrespective of time constraints (Irish and Piguet, 2013). Future work developing methods and materials that fully capture the depth and breadth of semantic memory and processing will be critical in facilitating comparison across forms of memory and in understanding their cognitive and neural (inter)dependencies as well as in testing the psychological and anatomical reality of the distinction in memory between semantic and episodic memory.

Integrating the study of episodic and semantic, understanding their interactions, interdependencies, and shared mechanisms, promises to advance our understanding of how words, concepts, and meaning, as well as episodes and events, are integrated, instantiated and maintained in memory, giving new insights into our two most quintessentially human abilities: memory and language.

## Author Contributions

MD and NJC planned the scope and content of the review. MD did the majority of the writing for the initial version of the manuscript with assistance from NVC and CH. All authors contributed to the final version of the manuscript, intellectually and in the writing and editing.

## Conflict of Interest

The authors declare that the research was conducted in the absence of any commercial or financial relationships that could be construed as a potential conflict of interest.

## References

[B1] Adelman J. S. Brown G. D. A. Quesada J. F. (2006). Contextual diversity, not word frequency, determines word-naming and lexical decision times. Psychol. Sci. 17, 814–823. 10.1111/j.1467-9280.2006.01787.x 16984300

[B200] Arnon I. Christiansen M. H. (2017). The role of multiword building blocks in explaining L1–L2 differences. Top. Cogn. Sci. 9, 621–636. 10.1111/tops.12271 28621472

[B201] Baddeley A. Vargha-Khadem F. Mishkin M. (2001). Preserved recognition in a case of developmental amnesia: implications for the acquisition of semantic memory? J. Cogn. Neurosci. 13, 357–369. 10.1162/08989290151137403 11371313

[B2] Baddeley A. D. Wilson B. A. (1994). When implicit learning fails: amnesia and problem of error elimination. Neuropsychologia 32, 53–68. 10.1016/0028-3932(94)90068-x 8818154

[B3] Bayley P. Squire L. (2002). Medial temporal lobe amnesia: gradual acquisition of factual information by nondeclarative memory. J. Neurosci. 22, 5741–5748. 10.1523/JNEUROSCI.22-13-05741.2002 12097527 PMC2821083

[B202] Bennett S. D. Burnett A. N. Siakaluk P. D. Pexman P. M. (2011). Imageability and body-object interaction ratings for 599 multisyllabic nouns. Behav. Res. Methods 43, 1100–1109. 10.3758/s13428-011-0117-5 21681627

[B4] Binder J. R. Desai R. H. (2011). The neurobiology of semantic memory. Trends Cogn. Sci. 15, 527–536. 10.1016/j.tics.2011.10.001 22001867 PMC3350748

[B5] Binder J. R. Desai R. H. Graves W. W. Conant L. L. (2009). Where is the semantic system? A critical review and meta-analysis of 120 functional neuroimaging studies. Cereb. Cortex 19, 2767–2796. 10.1093/cercor/bhp055 19329570 PMC2774390

[B6] Blumenthal A. Duke D. Bowles A. Gilboa R. Rosenbaum R. Kohler K.. (2017). Abnormal semantic knowledge as a case of developmental amnesia. Neuropsychologia 102, 237–247. 10.1093/cercor/bhp055 28625659

[B7] Bonhage C. Mueller L. Friederici A. Fiebach C. (2015). Combined eye tracking and fMRI reveals neural basis of linguistic predictions during sentence comprehension. Cortex 68, 33–47. 10.1016/j.cortex.2015.04.011 26003489

[B8] Brodeur M. B. Dionne-Dostie E. Montreuil T. Lepage M. (2010). The bank of standardized stimuli (BOSS), a new set of 480 normative photos of objects to be used as visual stimuli in cognitive research. PLoS One 5:e10773. 10.1371/journal.pone.0010773 20532245 PMC2879426

[B203] Brodeur M. B. Guérard K. Bouras M. (2014). Bank of standardized stimuli (BOSS) phase II: 930 new normative photos. PLoS One 9:e106953. 10.1371/journal.pone.0106953 25211489 PMC4161371

[B9] Buckner R. (2010). The role of the hippocampus in prediction and imagination. Annu. Rev. Psychol. 61, 27–48. 10.1146/annurev.psych.60.110707.163508 19958178

[B204] Buckner R. L. Carroll D. C. (2007). Self-projection and the brain. Trends Cogn. Sci. 11, 49–57. 10.1016/j.tics.2006.11.004 17188554

[B10] Buckner R. Carroll D. (2007). Self-projection and the brain. Trends Cogn. Sci. 11, 49–57. 10.1016/j.tics.2006.11.004 17188554

[B11] Chambers K. E. Onishi K. H. Fisher C. (2003). Infants learn phonotactic regularities from brief auditory experience. Cognition 87, B69–B77. 10.1016/s0010-0277(02)00233-0 12590043

[B12] Clarke A. Tyler L. (2015). Understanding what we see: how we derive meaning from vision. Trends Cogn. Sci. 19, 677–687. 10.1016/j.tics.2015.08.008 26440124 PMC4636429

[B205] Cohen N. J. (1984). “Preserved learning capacity in amnesia: evidence for multiple memory systems,” in Neuropsychology of Memory, eds Squire L. R. Butters N. (New York, NY: Guilford Press), 83–103.

[B13] Cohen N. J. Eichenbaum H. (1993). Memory, Amnesia and the Hippocampal System. Cambridge, MA: MIT Press.

[B15] Cohen N. J. Poldrack R. A. Eichenbaum H. (1997). Memory for items and memory for relations in the procedural/declarative memory framework. Memory 5, 131–178. 10.1080/741941149 9156097

[B14] Cohen N. J. Squire L. R. (1980). Preserved learning and retention of a pattern-analyzing skill in amnesia: dissociation of knowing how and knowing that. Science 210, 207–210. 10.1126/science.7414331 7414331

[B16] Corkin S. (2002). What’s new with the amnesic patient H.M.? Nat. Rev. Neurosci. 3, 153–160. 10.1038/nrn726 11836523

[B19] Covington N. V. Brown-Schmidt S. Duff M. C. (2018). The necessity of the hippocampus for statistical learning. J. Cogn. Neurosci. 30, 680–697. 10.1162/jocn_a_01228 29308986 PMC5876146

[B17] Covington N. Duff M. C. (2016). Expanding the language network: direct contributions from hippocampus. Trends Cogn. Sci. 20, 869–870. 10.1016/j.tics.2016.10.006 27814958 PMC5345935

[B18] Covington N. Kurczek J. Duff M. C. (2017). “Metaphor generation and comprehension in individuals with hippocampal amnesia,” in Poster Presentation at the American Speech Language Hearing Association (Los Angeles, CA, USA).

[B20] Cross Z. R. Kohler M. J. Schlesewsky M. Gaskell M. G. Bornkessel-Schlesewky I. (2018). Sleep-dependent memory consolidation and incremental sentence comprehension: computational dependencies during language learning as revealed by neuronal oscillations. Front. Hum. Neurosci. 12:18. 10.3389/fnhum.2018.00018 29445333 PMC5797781

[B21] Damasio A. R. Tranel D. Damasio H. (1989). “Amnesia caused by herpes simplex encephalitis, infarctions in basal forebrain, Alzheimer’s disease and anoxia/ischemia,” in Handbook of Neuropsychology (Vol. 3), eds Boller F. Grafman J. (Amsterdam: Elsevier), 149–166.

[B206] Davachi L. (2006). Item, context and relational episodic encoding in humans. Curr. Opin. Neurobiol. 16, 693–700. 10.1016/j.conb.2006.10.012 17097284

[B22] Davachi L. Mitchelle J. Wagner A. (2003). Mutliple routes to memory: distinct medial temporal lobe processes build item and source memories. Proc. Natl. Acad. Sci. U S A 100, 2157–2162. 10.1073/pnas.0337195100 12578977 PMC149975

[B23] Davies R. R. Graham K. S. Xuereb J. H. Williams G. B. Hodges J. R. (2004). The human perirhinal cortex and semantic memory. Eur. J. Neurosci. 20, 2441–2446. 10.1111/j.1460-9568.2004.03710.x 15525284

[B24] Davis M. Gaskell G. (2009). A complementary systems account of word learning: neural and behavioral evidence. Philos. Trans. R. Soc. Lond. B Biol. Sci. 364, 3773–3800. 10.1098/rstb.2009.0111 19933145 PMC2846311

[B25] Dawood M. Cole A. Hilverman C. Duff M. C. (2018). “Hippocampal contributions to remote semantic memory: evidence of impaired naming in amnesia,” in Poster Presentation at the American Speech Language Hearing Association (Boston, MA, USA).

[B207] Dewar M. Garcia Y. F. Cowan N. Della Sala S. (2009). Delaying interference enhances memory consolidation in amnesic patients. Neuropsychology 23, 627–634. 10.1037/a0015568 19702416 PMC2808210

[B26] Duff M. C. Hengst J. Tranel D. Cohen N. J. (2006). Development of shared information in communication despite hippocampal amnesia. Nat. Neurosci. 9, 140–146. 10.1038/nn1601 16341214

[B27] Duff M. C. Hengst J. Tranel D. Cohen N. J. (2009). Hippocampal amnesia disrupts verbal play and the creative use of language in social interaction. Aphasiology 23, 926–939. 10.1080/02687030802533748 20300442 PMC2840642

[B208] Duñabeitia J. A. Avilés A. Carreiras M. (2008). NoA’s ark: influence of the number of associates in visual word recognition. Psychon. Bull. Rev. 15, 1072–1077. 10.3758/pbr.15.6.1072 19001569

[B29] Eichenbaum H. (2017). On the integration of space, time, and memory. Neuron 95, 1007–1018. 10.1016/j.neuron.2017.06.036 28858612 PMC5662113

[B30] Eichenbaum H. Cohen N. J. (2001). From Conditioning to Conscious Recollection: Memory Systems of the Brain. New York, NY: Oxford University Press.

[B209] Eichenbaum H. B. (1998). Amnesia, the hippocampus, and episodic memory. Hippocampus 8:197. 10.1002/(sici)1098-1063(1998)8:3<205::aid-hipo3>3.0.co;2-i 9662133

[B31] Eichenbaum H. Yonelinas A. P. Ranganath C. (2007). The medial temporal lobe and recognition memory. Annu. Rev. Neurosci. 30, 123–152. 10.1146/annurev.neuro.30.051606.094328 17417939 PMC2064941

[B210] Ellis N. C. Ogden D. C. (2017). Thinking about multiword constructions: usage-based approaches to acquisition and processing. Topics Cogn. Sci. 9, 604–620. 10.1111/tops.12256 28233939

[B33] Elward R. L. Dzieciol A. M. Vargha-Khadem F. (2019). Little evidence for fast mapping in adults with developmental amnesia. Cogn. Neurosci. 10, 215–217. 10.1080/17588928.2019.1593123 30894071

[B32] Elward R. L. Vargha-Khadem F. (2018). Semantic memory in developmental amnesia. Neurosci. Lett. 680, 23–30. 10.1016/j.neulet.2018.04.040 29715544 PMC6086936

[B34] Ferreira C. Charest I. Wimber M. (2019). Retrieval aids the creation of a generalised memory trace and strengthens episode-unique information. Neuroimage 201:115996. 10.1016/j.neuroimage.2019.07.009 31280012

[B36] Gabrieli J. D. E. (1998). Cognitive neuroscience of human memory. Annu. Rev. Psychol. 49, 87–115. 10.1146/annurev.psych.49.1.87 9496622

[B35] Gabrieli J. D. E. Cohen N. J. Corkin S. (1988). The impaired learning of semantic knowledge following bilateral medial temporal-lobe resection. Brain Cogn. 7, 157–177. 10.1016/0278-2626(88)90027-9 3377896

[B211] Gardiner J. M. Brandt K. R. Baddeley A. D. Vargha-Khadem F. Mishkin M. (2008). Charting the acquisition of semantic knowledge in a case of developmental amnesia. Neuropsychologia 46, 2865–2868. 10.1016/j.neuropsychologia.2008.05.021 18589461 PMC2702517

[B37] Gaskell M. G. Cairney S. Rodd J. (2019). Contextual priming of word meanings is stabilized over sleep. Cognition 182, 109–126. 10.1016/j.cognition.2018.09.007 30227332

[B213] Glisky E. L. (1992). Acquisition and transfer of declarative and procedural knowledge by memory-impaired patients: a computer data-entry task. Neuropsychologia 30, 899–910. 10.1016/0028-3932(92)90034-j 1436436

[B38] Glisky E. L. Schacter D. L. Tulving E. (1986). Computer learning by memory-impaired patients: acquisition and retention of complex knowledge. Neuropsychologia 24, 313–328. 10.1016/0028-3932(86)90017-5 3755511

[B39] Gold J. Squire L. (2005). Quantifying medial temporal lobe damage in memory-impaired patients. Hippocampus 15, 79–85. 10.1002/hipo.20032 15390163 PMC2754383

[B49] Gordon Hayman C. A. Macdonald C. A. Tulving E. (1993). The role of repetition and associative interference in new semantic learning in amnesia: a case experiment. J. Cogn. Neurosci. 5, 375–389. 10.1162/jocn.1993.5.4.375 23964914

[B40] Gómez R. L. (2002). Variability and detection of invariant structure. Psychol. Sci. 13, 431–437. 10.1111/1467-9280.00476 12219809

[B41] Graf Estes K. Evans J. L. Alibali M. W. Saffran J. R. (2007). Can infants map meaning to newly segmented words? Statistical segmentation and word learning. Psychol. Sci. 18, 254–260. 10.1111/j.1467-9280.2007.01885.x 17444923 PMC3864753

[B42] Greenberg D. L. Verfaellie M. (2010). Interdependence of episodic and semantic memory: evidence from neuropsychology. J. Int. Neuropsychol. Soc. 16, 748–753. 10.1017/s1355617710000676 20561378 PMC2952732

[B43] Grilli M. D. Verfaellie M. (2014). Personal semantic memory: insights from neuropsychological research on amnesia. Neuropsychologia 61, 56–64. 10.1016/j.neuropsychologia.2014.06.012 24949553

[B214] Grondin R. Lupker S. J. McRae K. (2009). Shared features dominate semantic richness effects for concrete concepts. J. Mem. Lang. 60, 1–19. 10.1016/j.jml.2008.09.001 20046224 PMC2634287

[B44] Gupta Gordon R. Duff M. C. Cohen N. J. (2018). “Applications of collaborative memory: patterns of success and failure in individuals with hippocampal amnesia,” in Collaborative Remembering: How Remembering With Others Influences Memory, eds Meade M. Barnier A. Van Bergen P. Harris C. Sutton J..

[B45] Gupta P. (2012). “Word learning as the confluence of memory mechanisms: computational and neural evidence,” in The Handbook of the Neurospychology of Language, Vol. 1 (1st Edn.), ed. Faust M. (Chichester, England: Wiley-Blackwell), 146–163.

[B215] Hamann S. B. Squire L. R. (1995). On the acquisition of new declarative knowledge in amnesia. Behav. Neurosci. 109, 1027–1044. 10.1037/0735-7044.109.6.1027 8748954

[B46] Hamamé C. M. Alario F. X. Llorens A. Liégeois-Chauvel C. Trébuchon-Da Fonseca A. (2014). High frequency γ activity in the left hippocampus predicts visual object naming performance. Brain Lang. 135, 104–114. 10.1016/j.bandl.2014.05.007 25016093

[B47] Hannula D. Duff M. C. (Eds). (2017). The Hippocampus From Cells to Systems: Structure, Connectivity, and Functional Contributions to Memory and Flexible Cognition. Switzerland: Springer International Publishing.

[B216] Hannula D. E. Greene A. (2012). The hippocampus reevaluated in unconscious learning and memory: at a tipping point? Front. Hum. Neurosci. 6:80. 10.3389/fnhum.2012.00080 22518102 PMC3324888

[B217] Hannula D. E. Ranganath C. (2008). Medial temporal lobe activity predicts successful relational memory binding. J. Neurosci. 28, 116–124. 10.1523/JNEUROSCI.3086-07.2008 18171929 PMC2748793

[B48] Hannula D. Ryan J. Warren D. (2017). “Beyond long-term declarative memory: hippocampal contributions to preception, short term retention and unconscious memory expression,” in The Hippocampus from Cells to Systems: Structure, Connectivity, and Functional Contributions to Memory and Flexible Cognition, eds Hannula D. Duff M. C. (Springer International Publishing: Switzerland), 281–336.

[B218] Hannula D. E. Tranel D. Cohen N. J. (2006). The long and the short of it: relational memory impairments in amnesia, even at short lags. J. Neurosci. 26, 8352–8359. 10.1523/JNEUROSCI.5222-05.2006 16899730 PMC6673802

[B219] Hargreaves I. S. Pexman P. M. (2012). Does richness lose its luster? Effects of extensive practice on semantic richness in visual word recognition. Front. Hum. Neurosci. 6:234. 10.3389/fnhum.2012.00234 22912610 PMC3418682

[B220] Hargreaves I. S. Pexman P. M. Johnson J. S. Zdrazilova L. (2012). Richer concepts are better remembered: number of features effects in free recall. Front. Hum. Neurosci. 6:73. 10.3389/fnhum.2012.00073 22514526 PMC3322485

[B50] Henke K. (2010). A model for memory systems based on processing modes rather than consciousness. Nat. Rev. Neurosci. 11, 523–532. 10.1038/nrn2850 20531422

[B51] Heyworth N. Squire L. (2019). The nature of recollection across months and years after medial temporal lobe damage. Proc. Natl. Acad. Sci. U S A 116, 4619–4624. 10.1073/pnas.1820765116 30792351 PMC6410823

[B230] Hilverman C. Cook S. W. Duff M. C. (2016). Hippocampal declarative memory supports gesture production: evidence from amnesia. Cortex 85, 25–36. 10.1016/j.cortex.2016.09.015 27810497 PMC5127754

[B52] Hilverman C. Cook S. W. Duff M. C. (2017). Influence of hippocampal declarative memory on word use: patients with amnesia use less imageable words. Neuropsychologia 106, 179–186. 10.1016/j.neuropsychologia.2017.09.028 28970108 PMC5813699

[B54] Irish M. Eyre N. Dermody N. O’Callaghan C. Hodges J. R. Hornberger M.. (2016). Neural substrates of semantic prospection—evidence from the dementias. Front. Behav. Neurosci. 10:96. 10.3389/fnbeh.2016.00096 27252632 PMC4877391

[B53] Irish M. Piguet O. (2013). The pivotal role of semantic memory in remembering the past and imagining the future. Front. Behav. Neurosci. 7:27. 10.3389/fnbeh.2013.00027 23565081 PMC3615221

[B55] Jafarpour A. Piai V. Lin J. J. Knight R. T. (2017). Human hippocampal pre-activation predicts behavior. Sci. Rep. 7:5959. 10.1038/s41598-017-06477-5 28729738 PMC5519691

[B240] Katz A. N. (1989). On choosing the vehicles of metaphors: referential concreteness, semantic distances, and individual differences. J. Mem. Lang. 28, 486–499. 10.1016/0749-596x(89)90023-5

[B56] Keane M. Bousquet K. Wank A. Verfaeliie M. (2019). Relational processing in the semantic domain is impaired in medial temporal lobe amnesia. J. Neuropsychol. [Epub ahead of print]. 10.1111/jnp.12196 31729186 PMC7220829

[B57] Kensinger E. A. Ullman M. T. Corkin S. (2001). Bilateral medial temporal lobe damage does not affect lexical or grammatical processing: evidence from the amnesic patient H.M. Hippocampus 11, 347–360. 10.1002/hipo.1049.abs 11530839

[B250] Kinsbourne M. Wood F. (1975). “Short term memory processes and the amnesic syndrome,” in Short-Term Memory, eds Deutsch D. Deutsch J. S. (San Diego, CA: Academic Press), 258–291.

[B58] Klooster N. Duff M. C. (2015). Remote semantic memory is impoverished in hippocampal amnesia. Neuropsychologia 79, 42–52. 10.1016/j.neuropsychologia.2015.10.017 26474741 PMC4679630

[B59] Klooster N. Tranel D. Duff M. C. (2019). Hippocampus and semantic memory across time. Brain Lang. 201:104711. 10.1016/j.bandl.2019.104711 31739112 PMC7577377

[B60] Koutstaal W. (2019). Other ‘routes in’? Has the ‘fast’ in the fast mapping concept led us astray? Cogn. Neurosci. 10, 213–214. 10.1080/17588928.2019.1593124 30895846

[B61] Kovner R. Mattis S. Goldmeier E. (1983). A technique for promoting robust free recall in chronic organic amnesia. J. Clin. Neuropsychol. 5, 65–71. 10.1080/01688638308401151 6826765

[B260] Lakoff G. Johnson M. (1980). The metaphorical structure of the human conceptual system. Cogn. Sci. 4, 195–208. 10.1207/s15516709cog0402_4

[B270] Lambon Ralph M. Lowe C. Rogers T. (2007). Neural basis of category-specific semantic deficits for living things: evidence from semantic dementia, HSVE, and a neural network model. Brain 130, 1127–1137. 10.1093/brain/awm025 17438021

[B62] Lany J. (2014). Judging words by their covers and the company they keep: probabilistic cues support word learning. Child Dev. 85, 1727–1739. 10.1111/cdev.12199 24354917 PMC4450861

[B63] Lany J. Saffran J. R. (2011). Interactions between statistical and semantic information in infant language development. Dev. Sci. 14, 1207–1219. 10.1111/j.1467-7687.2011.01073.x 21884336 PMC3867528

[B280] Levine B. Svoboda E. Hay J. F. Winocur G. Moscovitch M. (2002). Aging and autobiographical memory: dissociating episodic from semantic retrieval. Psychol. Aging 17, 677–689. 10.1037/0882-7974.17.4.677 12507363

[B64] Lucas H. D. Duff M. C. Cohen N. J. (2019). The hippocampus promotes effective saccadic information gathering in humans. J. Cogn. Neurosci. 31, 186–201. 10.1162/jocn_a_01336 30188777

[B290] Lucas H. D. Hubbard R. J. Federmeier K. D. (2017). Flexible conceptual combination: electrophysiological correlates and consequences for associative memory. Psychophysiology 54, 833–847. 10.1111/psyp.12840 28191647 PMC5611855

[B300] Makkai A. Boatner T. Gates J. E. (1995). Dictionary of American Idioms. Los Angeles, CA: Barrons International.

[B68] Manns J. R. (2004). J.F.K., L.B.J., and H.M.: the famous memories of a famous amnesic. Hippocampus 14, 411–412. 10.1002/hipo.20010 15224977

[B67] Manns J. R. Hopkins R. O. Squire L. R. (2003). Semantic memory and the human hippocampus. Neuron 38, 127–133. 10.1016/s0896-6273(03)00146-6 12691670

[B69] Martin A. Chao L. L. (2001). Semantic memory and the brain: structure and processes. Curr. Opin. Neurobiol. 11, 194–201. 10.1016/s0959-4388(00)00196-3 11301239

[B400] McClelland J. L. McNaughton B. L. O’Reilly R. C. (1995). Why there are complementary learning systems in the hippocampus and neocortex: insights from the successes and failures of connectionist models of learning and memory. Psychol. Rev. 102, 419–457. 10.1037/0033-295x.102.3.419 7624455

[B500] McGlone M. S. Glucksberg S. Cacciari C. (1994). Semantic productivity and idiom comprehension. Discourse Process. 17, 167–190. 10.1080/01638539409544865

[B71] Meeter M. Murre J. (2004). Consolidation of long-term memory: evidence and alternatives. Psychol. Bull. 130, 843–857. 10.1037/0033-2909.130.6.843 15535740

[B600] Mishkin M. Vargha-Khadem F. Gadian D. G. (1998). Amnesia and the organization of the hippocampal system. Hippocampus 8, 212–216. 10.1002/(sici)1098-1063(1998)8:3<212::aid-hipo4>3.0.co;2-l 9662136

[B72] Mollica F. Piantadosi S. T. (2019). Humans store about 1.5 megabytes of information during language acquisition. R. Soc. Open Sci. 6:181393. 10.1098/rsos.181393 31032001 PMC6458406

[B73] Moscovitch M. Cabeza R. Winocur G. Nadel L. (2016). Episodic memory and beyond: the hippocampus and neocortex in transformation. Annu. Rev. Psychol. 67, 105–134. 10.1146/annurev-psych-113011-143733 26726963 PMC5060006

[B700] Moscovitch M. Nadel L. Winocur G. Gilboa A. Rosenbaum R. S. (2006). The cognitive neuroscience of remote episodic, semantic and spatial memory. Curr. Opin. Neurobiol. 16, 179–190. 10.1016/j.conb.2006.03.013 16564688

[B74] Nadel L. (1991). The hippocampus and space revisited. Hippocampus 1, 221–229. 10.1002/hipo.450010302 1669368

[B75] Nadel L. Moscovitch M. (1997). Memory consolidation, retrograde amnesia and the hippocampal complex. Curr. Opin. Neurobiol. 7, 217–227. 10.1016/s0959-4388(97)80010-4 9142752

[B77] O’Kane G. Kensinger E. A. Corkin S. (2004). Evidence for semantic learning in profound amnesia: an investigation with patient H.M. Hippocampus 14, 417–425. 10.1002/hipo.20005 15224979

[B76] O’Keefe J. Nadel L. (1978). The Hippocampus as a Cognitive Map. Oxford: Clarendon Press.

[B800] Olson I. R. Page K. Moore K. S. Chatterjee A. Verfaellie M. (2006). Working memory for conjunctions relies on the medial temporal lobe. J. Neurosci. 26, 4596–4601. 10.1523/JNEUROSCI.1923-05.2006 16641239 PMC1764465

[B79] Pacton S. Fayol M. Perruchet P. (2005). Children’s implicit learning of graphotactic and morphological regularities. Child Dev. 76, 324–339. 10.1111/j.1467-8624.2005.00848.x 15784085

[B900] Pawley A. Syder F. H. (1980). “Two puzzles for linguistic theory: nativelike selection and nativelike fluency,” in Communicative Competence, eds Richards J. C. Schmidt R. (London: Longmans), 191–225.

[B901] Pexman P. M. Holyk G. G. Monfils M. H. (2003). Number-of-features effects and semantic processing. Mem. Cognit. 31, 842–855. 10.3758/bf03196439 14651293

[B902] Pexman P. M. Lupker S. J. Hino Y. (2002). The impact of feedback semantics in visual word recognition: number-of-features effects in lexical decision and naming tasks. Psychon. Bull. Rev. 9, 542–549. 10.3758/bf03196311 12412895

[B81] Piai V. Anderson K. Lin J. Dewar C. Parvizi J. Dronkers N.. (2016). Direct brain recordings reveal hippocampal rhythm underpinnings of language processing. Proc. Natl. Acad. Sci. U S A 113, 11366–11371. 10.1073/pnas.1603312113 27647880 PMC5056038

[B82] Postle B. R. Corkin S. (1998). Impaired word-stem completion priming but intact perceptual identification priming with novel words: evidence from the amnesic patient H.M. Neuropsychologia 36, 421–440. 10.1016/s0028-3932(97)00155-3 9699950

[B903] Race E. Keane M. M. Verfaellie M. (2011). Medial temporal lobe damage causes deficits in episodic memory and episodic future thinking not attributable to deficits in narrative construction. J. Neurosci. 31, 10262–10269. 10.1523/JNEUROSCI.1145-11.2011 21753003 PMC4539132

[B83] Ranganath C. (2010). A unified framework for the functional organization of the medial temporal lobes and the phenomenology of episodic memory. Hippocampus 20, 1263–1290. 10.1002/hipo.20852 20928833

[B84] Reber P. Knowlton B. Squire L. (1996). Dissociable properties of memory systems: differences in the flexibility of declarative and nondeclarative knowledge. Behav. Neurosci. 110, 861–871. 10.1037/0735-7044.110.5.861 8918990

[B85] Reilly J. Peelle J. E. Garcia A. Crutch S. J. (2016). Linking somatic and symbolic representation in semantic memory: the dynamic multilevel reactivation framework. Psychon. Bull. Rev. 23, 1002–1014. 10.3758/s13423-015-0824-5 27294419 PMC5156531

[B904] Renoult L. Irish M. Moscovitch M. Rugg M. D. (2019). From Knowing to Remembering: the Semantic-Episodic distinction. Trends Cogn. Sci. 23, 1041–1057. 10.1016/j.tics.2019.09.008 31672430

[B86] Ritchey M. Libby L. Ranganath C. (2015). Cortico-hippocampal systems involved in memory and cognition: the PMAT framework. Prog. Brain Res. 219, 45–64. 10.1016/bs.pbr.2015.04.001 26072233

[B905] Robin J. Rivest J. Rosenbaum R. S. Moscovitch M. (2019). Remote spatial and autobiographical memory in cases of episodic amnesia and topographical disorientation. Cortex 119, 237–257. 10.1016/j.cortex.2019.04.013 31163312

[B87] Rogers T. T. Lambon Ralph M. A. Garrard P. Bozeat S. McClelland J. L. Hodges J. R.. (2004). Structure and deterioration of semantic memory: a neuropsychological and computational investigation. Psychol. Rev. 111, 205–235. 10.1037/0033-295x.111.1.205 14756594

[B89] Rosenbaum R. S. Gilboa A. Levine B. Winocur G. Moscovitch M. (2009). Amnesia as an impairment of detail generation and binding: evidence from personal, fictional, and semantic narratives in K.C. Neuropsychologia 47, 2181–2187. 10.1016/j.neuropsychologia.2008.11.028 19100757

[B90] Rosenbaum R. S. Kohler S. Schacter D. Moscovitch M. Westmacott R. Black S.. (2005). The case of K.C.: contributions of a memory-impaired person to memory theory. Neuropsychologia 43, 989–1021. 10.1016/j.neuropsychologia.2004.10.007 15769487

[B88] Rosenbaum R. S. Moscovitch M. Foster J. K. Schnyer D. M. Gao F. Kovacevic N.. (2008). Patterns of autobiographical memory loss in medial-temporal lobe amnesic patients. J. Cogn. Neurosci. 20, 1490–1506. 10.1162/jocn.2008.20105 18303977

[B91] Rubin R. D. Schwarb H. Lucas H. D. Dulas M. R. Cohen N. J. (2017). Dynamic hippocampal and prefrontal contributions to memory processes and representations blur the boundaries of traditional cognitive domains. Brain Sci. 7:82. 10.3390/brainsci7070082 28704928 PMC5532595

[B92] Rugg M. D. Johnson J. D. Uncapher M. R. (2015). “Encoding and retrieval in episodic memory: insights from fMRI,” in Handbook on the Cognitive Neuroscience of Memory, eds Duarte A. Barense M. Addis D. R. (Oxford, UK: Wiley-Blackwell), 84–107.

[B93] Ryan J. D. Althoff R. R. Whitlow S. Cohen N. J. (2000). Amnesia is a deficit in relational memory. Psychol. Sci. 11, 454–461. 10.1111/1467-9280.00288 11202489

[B94] Ryan L. Cox C. Hayes S. Nadel L. (2008). Hippocampal activation during episodic and semantic memory retrieval: comparing category production and category cured recall. Neuropsychologia 46, 2109–2121. 10.1016/j.neuropsychologia.2008.02.030 18420234 PMC2482601

[B96] Saffran J. R. Aslin R. N. Newport E. L. (1996). Statistical learning by 8-month-old infants. Science 274, 1926–1928. 10.1126/science.274.5294.1926 8943209

[B95] Saffran J. R. Wilson D. P. (2003). From syllables to syntax: multilevel statistical learning by 12-month-old infants. Infancy 4, 273–284. 10.1207/s15327078in0402_07

[B97] Schacter D. L. Addis D. R. (2007). The cognitive neuroscience of constructive memory: remembering the past and imagining the future. Philos. Trans. R. Soc. Lond. B Biol. Sci. 362, 773–786. 10.1098/rstb.2007.2087 17395575 PMC2429996

[B98] Schacter D. L. Addis D. R. Buckner R. (2008). Episodic simulation of future events: concepts, data, and applications. Ann. N Y Acad. Sci. 1124, 39–60. 10.1196/annals.1440.001 18400923

[B99] Schapiro A. C. Gregory E. Landau B. McCloskey M. Turk-Browne N. B. (2014). The necessity of the medial temporal lobe for statistical learning. J. Cogn. Neurosci. 26, 1736–1747. 10.1162/jocn_a_00578 24456393 PMC4264662

[B100] Schapiro A. C. Kustner L. V. Turk-Browne N. B. (2012). Shaping of object representations in the human medial temporal lobe based on temporal regularities. Curr. Biol. 22, 1622–1627. 10.1016/j.cub.2012.06.056 22885059 PMC3443305

[B906] Sharon T. Moscovitch M. Gilboa A. (2011). Rapid neocortical acquisition of long-term arbitrary associations independent of the hippocampus. Proc. Natl. Acad. Sci. U S A 108, 1146–1151. 10.1073/pnas.1005238108 21199935 PMC3024695

[B101] Schlichting M. L. Preston A. R. (2015). Memory integration: neural mechanisms and implications for behavior. Curr. Opin. Behav. Sci. 1, 1–8. 10.1016/j.cobeha.2014.07.005 25750931 PMC4346341

[B102] Schmolck H. Kensinger E. Corkin S. Squire L. (2002). Semantic knowledge in patient H.M. and other patients with bilateral medial and lateral temporal lobe lesions. Hippocampus 12, 520–533. 10.1002/hipo.10039 12201637

[B103] Scoville W. B. Milner B. (1957). Loss of recent memory after bilateral hippocampal lesions. J. Neurol. Neurosurg. Psychiatry 20, 11–12. 10.1136/jnnp.20.1.11 13406589 PMC497229

[B105] Shimamura A. P. Squire L. (1984). Paired-associate learning and priming effects in amnesia: a neuropsychological study. J. Exp. Psychol. Gen. 113, 556–570. 10.1037/0096-3445.113.4.556 6240522

[B106] Skotko B. Kensinger E. Locascio J. Einstein G. Rubin D. Tupler L.. (2004). Puzzling thoughts for H.M.: can new semantic information be anchored to old semantic memories? Neuropsychology 18, 756–769. 10.1037/0894-4105.18.4.756 15506844

[B107] Solomon E. A. Lega B. C. Sperling M. R. Kahana M. J. (2019). Hippocampal theta codes for distances in semantic and temporal spaces. Proc. Natl. Acad. Sci. U S A 116, 24343–24352. 10.1073/pnas.1906729116 31723043 PMC6883851

[B108] Squire L. R. (1992). Memory and the hippocampus: a synthesis from findings with rats, monkeys and humans. Psychol. Rev. 99, 195–231. 10.1037/0033-295x.99.2.195 1594723

[B907] Squire L. R. Zola S. M. (1996). Structure and function of declarative and nondeclarative memory systems. Proc. Natl. Acad. Sci. U S A 93, 13515–13522. 10.1073/pnas.93.24.13515 8942965 PMC33639

[B908] Squire L. R. Zola S. M. (1998). Episodic memory, semantic memory, and amnesia. Hippocampus 8, 205–211. 10.1002/(sici)1098-1063(1998)8:3<205::aid-hipo3>3.0.co;2-i 9662135

[B110] Staresina B. Davachi L. (2009). Mind the gap: binding experiences across space and time in the human hippocampus. Neuron 63, 267–276. 10.1016/j.neuron.2009.06.024 19640484 PMC2726251

[B909] Stark S. M. Stark C. E. Gordon B. (2005). New semantic learning and generalization in an amnesic patient. Neuropsychology 19, 139–151. 10.1037/0894-4105.19.2.139 15769198

[B111] St-Laurent M. Moscovitch M. Jadd R. Mcandrews M. P. (2014). The perceptual richness of complex memory episodes is compromised by medial temporal lobe damage. Hippocampus 24, 560–576. 10.1002/hipo.22249 24449286

[B910] Taler V. Kousaie S. López Zunini R. (2013). ERP measures of semantic richness: the case of multiple senses. Front. Hum. Neurosci. 7:5. 10.3389/fnhum.2013.00005 23386817 PMC3560374

[B112] Thompson-Schill S. (2003). Neuroimaging studies of semantic memory: inferring “how” from “where”. Neuropsychologia 41, 280–292. 10.1016/s0028-3932(02)00161-6 12457754

[B911] Tulving E. (1972). “Episodic and semantic memory,” in Organization of Memory, eds Tulving E. Donaldson W. (New York, NY: Academic Press), 381–403.

[B912] Tulving E. Markowitsch H. J. (1998). Episodic and declarative memory: role of the hippocampus. Hippocampus 8, 198–204. 10.1002/(sici)1098-1063(1998)8:3<198::aid-hipo2>3.0.co;2-g 9662134

[B113] Tulving E. Hayman C. A. Macdonald C. A. (1991). Long-lasting perceptual priming and semantic learning in amnesia: a case experiment. J. Exp. Psychol. Learn. Mem. Cogn. 17, 595–617. 10.1037/0278-7393.17.4.595 1832430

[B115] Vargha-Khadem F. Gadian D. G. Watkins K. E. Connely A. Van Paesschen W. Mishkin M. (1997). Differential effects of early hippocampal pathology on episodic and semantic memory. Science 277, 376–380. 10.1126/science.277.5324.376 9219696

[B116] Verfaellie M. Bousquet K. Keane M. M. (2014). Medial temporal and neocortical contributions to remote memory for semantic narratives: evidence from amnesia. Neuropsychologia 61, 105–112. 10.1016/j.neuropsychologia.2014.06.018 24953960 PMC4122606

[B117] Verfaellie M. Koseff P. Alexander M. P. (2000). Acquisition of novel semantic information in amnesia: effects of lesion location. Neuropsychologia 38, 484–492. 10.1016/s0028-3932(99)00089-5 10683398

[B118] Wang J. Cohn N. J. Voss J. (2015). Covert rapid action-memory simulation (CRAMS): a hypothesis of hippocampal-prefrontal interactions for adaptive behavior. Neurobiol. Learn. Mem. 117, 22–33. 10.1016/j.nlm.2014.04.003 24752152 PMC4291298

[B119] Warren D. E. Duff M. C. (2014). Not so fast: hippocampal amnesia slows word learning despite successful fast mapping. Hippocampus 24, 920–933. 10.1002/hipo.22279 24719218 PMC4301585

[B120] Warren D. E. Duff M. C. (2019). Fast mappers, slow learners: word learning without hippocampus is slow and sparse irrespective of methodology. Cogn. Neurosci. 10, 210–212. 10.1080/17588928.2019.1593120 30898013 PMC6690757

[B121] Wilson B. Moffat N. (1983). “Rehabilitation of memory for everyday life,” in Everyday Memory: Actions and Absent-Mindedness, eds Harris J. E. Morris P. (London: Academic Press), 207–233.

[B913] Wojcik E. H. (2018). The development of lexical-semantic networks in infants and toddlers. Child Dev. Perspect. 12, 34–38. 10.1111/cdep.12252

[B914] Wojcik E. H. Saffran J. R. (2013). The ontogeny of lexical networks: toddlers encode the relationships amongst referents when learning novel words. Psychol. Sci. 24, 1898–1905. 10.1177/0956797613478198 23938274 PMC3865601

[B915] Wojcik E. H. Saffran J. R. (2015). Toddlers encode similarities among novel words from meaningful sentences. Cognition 138, 10–20. 10.1016/j.cognition.2015.01.015 25704579 PMC4366300

[B916] Yap M. J. Pexman P. M. Wellsby M. Hargreaves I. S. Huff M. (2012). An abundance of riches: cross-task comparisons of semantic richness effects in visual word recognition. Front. Hum. Neurosci. 6:72. 10.3389/fnhum.2012.00072 22529787 PMC3328122

[B123] Yonelinas A. P. (2013). The hippocampus supports high-resolution binding in the service of perception, working memory and long-term memory. Behav. Brain Res. 254, 34–44. 10.1016/j.bbr.2013.05.030 23721964 PMC3773061

[B124] Yonelinas A. P. Ranganath C. Ekstrom A. Wiltgen B. (2019). A contextual binding theory of episodic memory: systems consolidation reconsidered. Nat. Rev. Neurosci. 20, 364–375. 10.1038/s41583-019-0150-4 30872808 PMC7233541

[B125] Zacks J. M. Speer N. K. Reynolds J. R. (2009). Segmentation in reading and film comprehension. J. Exp. Psychol. Gen. 138, 307–327. 10.1037/a0015305 19397386 PMC8710938

[B126] Zacks J. M. Swallow K. M. (2007). Event segmentation. Curr. Dir. Psychol. Sci. 16, 80–84. 10.1111/j.1467-8721.2007.00480.x 22468032 PMC3314399

[B917] Zettersten M. Wojcik E. Benitez V. Saffran J. (2018). The company objects keep: linking referents together during cross-situational word learning. J. Mem. Lang. 99, 62–67. 10.1016/j.jml.2017.11.001 29503502 PMC5828251

